# Effect of Gender on Postoperative Outcome and Duration of Ventilation After Coronary Artery Bypass Grafting (CABG)

**DOI:** 10.7759/cureus.37717

**Published:** 2023-04-17

**Authors:** Hassan M Alamri, Turki O Alotaibi, Abdulhadi A Alghatani, Tariq F Alharthy, Albaraa M Sufyani, Abdulrahman M Alharthi, Abdulkarim A Mahmoud, Mohammed K Almahdi, Nabil Alama, Khalid E Al-Ebrahim

**Affiliations:** 1 Medicine, Faculty of Medicine, King Abdulaziz University, Jeddah, SAU; 2 Medicine, King Abdulaziz University Hospital, Jeddah, SAU; 3 Surgery, King Abdulaziz University, Jeddah, SAU

**Keywords:** grafting, bypass, females, surgery, artery, coronary

## Abstract

Introduction: The study assessed coronary artery bypass grafting (CABG) postoperative outcomes and associated factors in Saudi male and female patients. This was a retrospective cohort of patients who underwent CABG at the King Abdulaziz University Hospital (KAUH), Jeddah, Saudi Arabia, from January 2015 to December 2022.

Results: We included 392 patients, of whom 63 (16.1%) were female. Female undergoing CABG were significantly older (p=0.0001), had a significantly higher incidence of diabetes (p=0.0001), obesity (p=0.001), hypertension (p=0.001), and congestive heart failure (p=0.005), with a smaller body surface area (BSA) (p=0.0001) than male. Though renal dysfunction, previous cerebrovascular accident/transient ischemic attack (CVA/TIA), and myocardial infarction (MI), incidences were similar in both genders. Females were at significantly higher risk of mortality (p=0.0001), longer hospital stay (p=0.0001), and prolonged ventilation (p=0.0001). Preoperative renal dysfunction was the only statistically significant predictor of postoperative complications (p=0.0001). Female gender and preoperative renal dysfunction, were significant independent predictors of postoperative mortality and prolonged ventilation (p=0.005).

Conclusion: This study’s findings indicated that females have worse CABG outcomes and a higher risk of morbidities and complications. Uniquely our study showed a higher incidence of prolonged ventilation in females postoperatively.

## Introduction

Cardiovascular disease (CVD) continues to be the leading cause of death in females. More females than males died from CVD between 1984 and 2012 [1]. Females have unique CVD risk factors, including gestational diabetes, premature births, autoimmune diseases, and treatment for breast cancer. Though diabetes and depression also affect males, they might be greater risk factors in females than in males [1-3]. The earliest data available were based on randomized controlled trials that assessed results only in males [4-5]. Studies showed that females have lower incidence of coronary artery disease than male (about 13%-16% in the late 1970s to 29% vs. 60% in male in 2014) [6-8]. As a result, few data have been used to guide therapy choices for females with coronary artery disease, which may not be applicable, appropriate, or ideal [8]. While some studies reported higher mortality postoperatively in females who undergo coronary artery bypass grafting (CABG) than males [9-11], some other studies showed no significant gender differences in CABG outcomes [12-15]. Propensity-matched comparisons have demonstrated no difference in postoperative mortality between matched pairs of female and male [16-17]. Females commonly present with CVD at an older age than male by 10 years and are prone to have symptoms of atypical angina, silent MI, and sudden death. They are more likely to have more risk factors and comorbidities and be admitted to hospital emergency units than males. However, they are less likely to undergo surgery or procedures and take medications than males [12-15]. Studying and evaluating postoperative outcomes in both genders, particularly females, after undergoing major surgery like CABG is critical to improve care and outcome. Therefore, this study aims to assess CABG postoperative outcomes and predict risk factors.

## Materials and methods

Study design and settings

This was a retrospective cohort study conducted at the King Abdulaziz University Hospital (KAUH), Jeddah, Saudi Arabia, from 1 January 2015 to December 2022. Some 392 patients underwent isolated coronary bypass surgery, of which females were 63 (16.1%). We excluded patients under 18 and over 75 years of age, reoperations and combined valvular or congenital heart surgery. All patients received left internal mammary artery and standardized technique of cardiopulmonary bypass, myocardial preservation, and coronary anastomosis. Strict protocols and uniform pathways were applied to transfusion, inotropes, extubation, intensive care, hospital management, and discharge. Postoperative myocardial infarction was diagnosed with new Q waves in two leads or more, increased creatinine kinase more than 700 U/L, or new regional wall abnormalities. Renal dysfunction was defined as an increase in serum creatinine levels of more than 1 mg/dL. Prolonged ventilation is defined as inability to be extubed after 24 h postoperatively. Vascular complications included groin hematoma or retroperitoneal hemorrhage requiring transfusion.

Data collection and management

Data collected were recorded in the Microsoft Excel Sheet, stored at the principal investigator’s office, and accessed only by the author or co-authors. Confidentiality was ensured by the anonymity of data collected since no identifying details were collected.

Statistical analysis

Data analysis was performed using the Statistical Package for the Social Sciences (SPSS) software (Version 24, IBM Corp., Armonk, NY). Categorical and numerical variables were presented as frequencies and percentages, while continuous variables were presented as a mean with standard deviation. Inferential statistics such as independent sample t-test, one-way analysis of variance (ANOVA), and Pearson correlation were compared to determine any correlations between variables. Logistic regression analysis was used to determine independent predictors of outcomes. Confidence intervals (CIs) and odds ratios were calculated with a p-value < 0.05 considered for statistical significance.

## Results

We included 392 patients, of whom 16.1% were females undergoing CABG. The preoperative risk profiles differed significantly between males and females (Table 1). Compared with males, females undergoing CABG were older (58.7 ± 9.7 years vs. 57.2 ± 10.3 years, p = 0.0001), had a higher incidence of diabetes (87.3% vs. 63.8%, p=0.0001), were more obese (7.9% vs. 1.5%, p=0.001), hypertensive (84.1% vs. 64.7%, p=0.001), and had a great incidence of congestive heart failure (42.9% vs. 27.4%, p=0.005) than males. Females undergoing CABG had a smaller body surface area (BSA) (1.7 ± 0.2 m2 vs. 1.8 ± 0.0.2 m2, p=0.0001). An elevated incidence of previous renal dysfunction, previous cerebrovascular accident/transient ischemic attack (CVA/TIA), and previous myocardial infarction (MI) were similar for males and females. The observed difference in mortality between females and males was highly significant (22.2% vs. 8.2%, p=0.0001) (Table 2).

**Table 1:** Preoperative characteristics of participants. COPD, chronic obstructive pulmonary disease; Cr, creatinine; CVA, cerebrovascular accident; IABP, intraaortic balloon pump; IV, intravenous; LAD, left anterior descending coronary artery; LM, left main; MI, myocardial infarction; STS, Society of Thoracic Surgeons; TIA, transient ischemic attack

Characteristics	Men	Women	p-value
No. of patients	329	63	
Saudi	87.2%	74.6%	0.01
Age (years)	57.2 ± 10.3	58.70 ± 9.7	0.0001
Morbid obesity	1.5%	7.9%	0.003
Diabetes	63.8%	87.3%	0.0001
Hypertension	64.7%	84.1%	0.003
Hypercholesterolemia	31.6%	20.6%	0.081
Renal dysfunction Cr > 2	6.1%	6.3%	0.935
Carotid vascular disease	4.6%	1.6%	0.275
Previous CVA/TIA	4.6%	4.8%	0.944
Previous MI	63.8%	58.7%	0.442
Smoking	36.2%	9.5%	0.0001
COPD	0.6%	1.6%	0.414
Peripheral vascular disease	1.8%	9.5%	0.001
Congestive heart failure	27.4%	42.9%	0.014
Body surface area (m^2^)	1.8 ± 0.2	1.7 ± 0.2	0.0001
Preoperative hematocrit	39.8 ± 8.1	33.7 ± 5.9	0.0001
LM or LAD > 50%	93.3%	96.8%	0.287
Preoperative			
IABP	3.3%	6.3%	0.255
Aspirin	89.1%	84.1%	0.265
IV nitroglycerin	51.4%	55.6%	0.542
IV heparin	54.7%	65.1%	0.128
Circumstance			
Elective	60.2%	42.9%	0.011
Urgent	20.7%	34.9%	0.014
Emergent	18.5%	22.2%	0.494

**Table 2:** Postoperative outcomes. COPD, chronic obstructive pulmonary disease; Cr, creatinine; CVA, cerebrovascular accident; IABP, intraaortic balloon pump; IV, intravenous; LAD, left anterior descending coronary artery; LM, left main; MI, myocardial infarction; STS, Society of Thoracic Surgeons; TIA, transient ischemic attack

Characteristics	Men		Women		p value		
Death	8.2%		22.2%		0.0001		
MI	7.6%		11.1%		0.351		
Inotropic support	32.2%		47.6%		0.019		
IABP	10.3%		17.5%		0.104		
CVA/TIA	2.7%		1.6%		0.596		
Renal	9.7%		14.3%		0.279		
Pulmonary	18.8%		17.5%		0.796		
Vascular	4.6%		12.7%		0.012		
Mesenteric [P1]	1.2%		7.9%		0.0001		
Bleeding	18.8%		20.6%		0.741		
Arrhythmia	13.4%		15.9%		0.598		
Sternal wound infection	7.0%		12.7%		0.124		
Leg infection	1.5%		11.1%		0.0001		
Length of stay (days)	10.1 ± 12.3		12.1 ± 12.4		0.0001		
Length of ventilation (in hours)		17.2 ± 30.3		44.5 ± 94.3		0.0001	
Duration of ventilation (>24 hours)		12.2%		31.7%		0.0001	

The incidence of MI, IABP, CVA/TIA, postoperative renal dysfunction, bleeding, arrhythmia, and sternal wound infection, comparing females with males, are not statistically significant as p=0.05. Length of hospital stay was also prolonged in females (12.1 ± 12.4 days vs. 10.1 ± 12.3 days, p=0.0001). The observed difference in prolonged ventilation (>24 h) between females and males was statistically highly significant (31.7% vs. 12.2%, p=0.0001) (Table 2).

Only sternal wound infection was a statistically significant predictor for length of hospital stay (p=0.0001) (Table 3).

**Table 3:** Independent predictors of length of hospital stay. R2 = 0.24; B coeff, β coefficient; COPD, chronic obstructive pulmonary disease; CVA, cerebrovascular accident; Prob, p value; SE, standard error of coefficient; TIA, transient ischemic attack

Variables	B coef	SE of beta	Prob
Preoperative variables			
Female gender	0.933	1.714	0.587
Previous CVA/TIA	1.863	2.936	0.526
Hypertension	1.429	1.360	0.294
COPD	6.836	7.342	0.352
Hypercholesterolemia	-0.474	1.351	0.726
Renal dysfunction	-0.947	2.873	0.742
Carotid vascular disease	-3.687	3.125	0.239
Postoperative variables (Complications)			
Pulmonary	0.122	1.683	0.942
Vascular	-1.916	2.946	0.516
Renal	3.750	2.427	0.123
CVA/TIA	0.076	4.030	0.985
Sternal wound infection	10.237	2.285	0.000
Arrhythmia	-0.002	1.903	0.999

Preoperative renal dysfunction was the only statistically significant predictor of postoperative complications (p=0.0001). Female gender (p=0.001), age (p=0.034), and preoperative renal dysfunction (p=0.0001) were significant independent predictors of postoperative death. Female gender, preoperative renal dysfunction, vascular disease, arrhythmia, and bleeding were significant predictors for prolonged ventilation of more than 24 h (p=0.005) (Table 4).

**Table 4:** Independent predictors of postoperative outcomes. c-statistic, 0.56; OR, odd ratio; CI, confidence interval; SE, standard error; Coef., logistic regression coefficient

Variables	Coef.	SE	p-value	OR [P1]	95% CI
Independent predictors of postoperative complications					
Female gender	-0.462	0.454	0.308	0.630	0.259-1.533
Age	0.017	0.018	0.360	1.017	0.981-1.054
Preoperative renal dysfunction	-3.068	0.476	<0.001	0.047	0.018-0.118
Independent predictors of postoperative death					
Female gender	-1.206	0.379	0.001	0.299	0.142-0.629
Age	0.037	0.017	0.034	1.037	1.003-1.073
Preoperative renal dysfunction	-1.885	0.480	<0.001	0.152	0.059-0.389
Independent predictors of prolonged ventilation (>24 hours)					
Female gender	-1.228	0.357	0.001	0.293	0.145-0.589
Renal dysfunction Cr > 2	-1.149	0.528	0.030	0.317	0.113-0.892
Vascular	-1.132	0.529	0.032	0.323	0.114-0.909
Arrhythmia	-1.317	0.377	<0.001	0.268	0.128-0.561
Bleeding	1.064	0.336	0.002	2.898	1.501-5.596

## Discussion

The CABG is the most common and conventional cardiac revascularization to improve myocardial perfusion [18]. This procedure is invasive, and its outcomes and complications may be affected by multiple factors. Our findings showed that female patients were older and had more comorbidities resulting in a worse outcome. Postoperatively, female patients required more inotropic support, prolonged ventilation, and longer hospital stays. Sternal wound infection was a predictor of length of hospital stay; whereas, females had, preoperative renal dysfunction, vascular disease, arrhythmia, and bleeding which were significant predictors for prolonged ventilation. Preoperative renal dysfunction was the only statistically significant determinant of postoperative complications. Female gender, age, and preoperative renal dysfunction were predictors of postoperative death. Our findings support previous studies. A previously reported study found that early mortality rates after OPCAB (off-pump coronary artery bypass) surgery in both genders were not significant, while 120-day mortality risk was significantly (p=0.026) lower in female [11]. However, our findings contrast some other studies. A study conducted by Mack et al. [19] found that female patients had a 73.3% higher mortality risk and 47% higher risk of bleeding than male patients after CABG surgery. These findings also agree with another study that evaluated CABG outcomes between 1999 and 2014 reported a higher 30-day mortality and 1-year mortality in female [8]. The high risk of preoperative comorbidities and postoperative complications in females may explain the high mortality risk. A cohort study of 6,250 patients reported a higher risk of comorbidities, including diabetes, hypertension, obesity, and heart failure, in female patients [20-22], which aligns with our study’s findings. This study also showed that female patients were more likely to sustain a stroke, cardiac arrest, renal failure, heart block, and sternal infection and needed more ventilation postoperatively than males. Our results concerning Saudi females undergoing cardiac surgery, as well as, other studies have confirmed the universal problem of higher mortality and morbidity in females [23]. We attributed the gender difference to several factors, including smaller coronaries, leading to incomplete revascularization and less utilization of internal mammary arteries. Every patient in our study, including females, received one internal mammary artery. Atypical clinical findings resulted in a delayed diagnosis and referral. Non-ideal body weight, height, and body mass index complicate the situation. A higher incidence of diabetes, psychological disturbances, especially depression worsen the prognosis. Other female risk factors including osteoporosis and hormonal differences affect sternal bone healing, increasing the risk of sternal wound infection. Preventive measures are crucial to avoid this complication [24]. Less favorable aspirin effect is reported in females, increasing thrombogenicity. The study also showed that the most common complications in females include increased perioperative myocardial infarction and respiratory dysfunction, leading to prolonged ventilation. Smoking, disproportionate body to lung mass index, complications, and emotional liability contribute to prolonged ventilation postoperatively. Reviewing the literature, several studies have shown promising results with catheter interventions for coronary and valve procedures in females [25-27]. Further, research and health education are warranted to identify risk factors for this gender bias in order to institute medical and surgical interventions to mitigate this problem. Strict protocols and guidelines are followed and enforced to minimize certain complications like acute kidney injury, exploration for postoperative bleeding, and unexpected emergency readmission after discharge [28-30]. Prospective randomized studies are necessary to compare the results of interventional coronary procedures to conventional surgery in females. In patients 60 years and older only septuagenarian women have an observed higher 30- and 180-day mortality risk after CABG surgery compared with men. Essential predictive risk factors for 30-day mortality are the use of the LIMA graft, perioperative MI and the prevalence of postoperative pneumonia, but not female gender. However, after 180-days of follow-up our investigation concludes that female gender becomes an independent adverse risk factor for mortality associated with CABG. Given the associated conditions in women, future efforts to maximize the use of LIMA graft and reduce the occurrence of postoperative complications such as perioperative MI and pneumonia are necessary to further improve clinical outcomes. In view of our findings, decision for surgical revascularization should not be based on gender [31-32].

Limitation of the study

This study is retrospective with a small sample size. Recall bias, confounders, and limited determination of causation might have affected the results. Moreover, this was a single-center study that limits the findings' generalization to other healthcare facilities. Therefore, multicenter prospective research with a larger sample size is recommended.

## Conclusions

This study confirmed that females have a higher risk of comorbidities, postoperative complications, gender disparity, age factor, and mortality than males. Female gender and preoperative renal dysfunction are significant predictors for postoperative mortality and prolonged ventilation, while sternal wound infection was the only predictor for lengthier hospital stays. Uniquely our study showed prolonged ventilation in female postoperatively. These findings highlight worse outcomes in females than males, mainly directed at a patient's preoperative health status. Targeted follow-up and approach of female patients undergoing CABG to focus on minimizing pre and postoperative risk factors are recommended.
